# Peritoneal dialysis: why not?

**DOI:** 10.1590/2175-8239-JBN-2023-E001en

**Published:** 2023-04-03

**Authors:** Maria Claudia Cruz Andreoli, Claudia Totoli, Daniel Ribeiro da Rocha, Layon Silveira Campagnaro

**Affiliations:** 1 Universidade Federal de São Paulo, Fundação Oswaldo Ramos, Hospital do Rim, São Paulo, Brazil

Peritoneal Dialysis (PD) as an option for Renal Replacement Therapy (RRT) for end-stage Chronic Kidney Disease (CKD). It has the advantage of being a home-based, portable modality, and probably due to its continuous character, it preserves residual renal function (RRF) for longer^1^. Despite these aspects, its use is still low in Brazil. According to the 2021 Brazilian Dialysis Census, we have only 5.8% of the population on chronic dialysis submitted to PD^2^. Could this low prevalence be explained by unfavorable outcomes associated with this modality? This is not what the literature shows.

Vicentini and Ponce’s study^3^, published in this issue, compared outcomes in a cohort of incident patients on planned and urgent-onset PD and HD over a 5-year period. The authors found no difference in survival between the modalities, demonstrating the non-inferiority of PD in relation to HD in a Brazilian center. This finding is corroborated by other publications. In an analysis comparing incident dialysis patients in Canada eligible for both HD and PD, Wong et al. found no difference in mortality between both methods^4^. In a systematic review using propensity scores, which are commonly used in individuals from different treatment groups to achieve balance in the distribution of confounding factors, allowing direct estimation of causal effects of treatment, Elsayed et al showed that PD and HD provided equivalent survival benefits, and that reported differences in outcomes between treatments largely reflect a combination of factors that are unrelated to clinical efficacy^5^. Unfortunately, no Latin American studies were included in this meta-analysis, which demonstrates the relevance of carrying out studies similar to the one published here.

The risk of death between patients on HD and PD has long been compared^6^,^7^, but conclusions are generally limited by the difficulty (which will probably never be resolved) of conducting a prospective randomized controlled study, without modality selection bias. Patients without access to nephrological care and education about dialysis modalities in earlier stages of CKD will have a greater chance of initiating emergency HD therapy through a central venous catheter, which worsens their prognosis and represents a selection bias^8^,^9^,^10^. In their study, Vicentini and Ponce comment that the center where the study was carried out “has the exceptional feature of having a greater number of patients on PD than on HD, as PD is the preferred mode of dialysis therapy”. Thus, although the authors did not discriminate which patients had their start on dialysis planned or not, the admission bias for emergency HD of the most critically ill patients reported in other studies may have been partially corrected.

With regards to PD data in Brazil, the multicenter prospective cohort study: The Brazilian Peritoneal Dialysis Multicenter Study (BRAZPD) brought important information about practice patterns and outcomes in our settings, showing peritonitis rates, technique survival, and patient survival similar to cohorts from developed countries^11^. Regarding costs, an analysis of the Brazilian scenario was conducted by de Abreu et al. who, when comparing the total cost of dialysis therapy, which included direct medical costs, direct non-medical costs and indirect costs, showed that the total cost of PD was lower than that of HD^12^.

One aspect that should be highlighted in relation to PD is its benefit as initial dialysis therapy, due to the better correlation with the preservation of RRF when compared to HD^1^. Studies have shown that RRF is an important predictor of overall survival in patients on dialysis^13^. Some authors even showed lower mortality in the first years after starting RRT in patients on PD when compared to HD^14^,^15^. In the study by Vicentini and Ponce, the RRF was not evaluated during follow-up.

This better preservation of the RRF in PD has been increasingly valued in clinical practice. In fact, in recent years, the increasing use of incremental PD has been observed in incident patients on dialysis^16^. This is a strategy that adopts a prescription of therapy with a dose below the standardized one, considering the role of RRF in solute clearance and volume control. This strategy reduces the patient’s treatment burden, improves their quality of life, and reduces the economic and environmental impact of dialysis. Studies have shown similar survival and hospitalization rates when compared to the standard dialysis prescription, in addition to the potential benefits regarding the preservation of residual diuresis, therapy survival (preservation of the peritoneal membrane) and lower infection rates^17^. It is, therefore, a form of dialysis that allows a gradual adaptation of the patient to RRT, since the initial dialysis dose is smaller, and will be incremented over time, as RRF decreases.

The trajectory of dialysis-dependent patients with CKD should ideally be guided by a “life plan”, that is, be prepared considering, in addition to clinical conditions, the needs, expectations and convenience of the patient at each stage of your life. From this perspective, due to its performance and specificities, there is no reason why PD should not be part of the treatment options to be considered.
